# Platelet–Eosinophil Interactions As a Potential Therapeutic Target in Allergic Inflammation and Asthma

**DOI:** 10.3389/fmed.2017.00129

**Published:** 2017-08-08

**Authors:** Sajeel A. Shah, Clive P. Page, Simon C. Pitchford

**Affiliations:** ^1^Sackler Institute of Pulmonary Pharmacology, Institute of Pharmaceutical Science, King’s College London, London, United Kingdom

**Keywords:** platelets, eosinophils, asthma, P-selectin, allergy, IgE

## Abstract

The importance of platelet activation during hemostasis is well understood. An understanding of these mechanisms has led to the use of several classes of anti-platelet drugs to inhibit aggregation for the prevention of thrombi during cardiovascular disease. It is now also recognized that platelets can function very differently during inflammation, as part of their role in the innate immune response against pathogens. This dichotomy in platelet function occurs through distinct physiological processes and alternative signaling pathways compared to that of hemostasis (leading to platelet aggregation) and is manifested as increased rheological interactions with leukocytes, the ability to undergo chemotaxis, communication with antigen-presenting cells, and direct anti-pathogen responses. Mounting evidence suggests platelets are also critical in the pathogenesis of allergic diseases such as asthma, where they have been associated with antigen presentation, bronchoconstriction, bronchial hyperresponsiveness, airway inflammation, and airway remodeling in both clinical and experimental studies. In particular, platelets have been reported bound to eosinophils in the blood of patients with asthma and the incidence of these events increases after both spontaneous asthma attacks in a biphasic manner, or after allergen challenge in the clinic. Platelet depletion in animal models of allergic airway inflammation causes a profound reduction in eosinophil recruitment to the lung, suggesting that the association of platelets with eosinophils is indeed an important event during eosinophil activation. Furthermore, in cases of severe asthma, and in animal models of allergic airways inflammation, platelet–eosinophil complexes move into the lung through a platelet P-selectin-mediated, eosinophil β1-integrin activation-dependent process, while platelets increase adherence of eosinophils to the vascular endothelium *in vitro*, demonstrating a clear interaction between these cell types in allergic inflammatory diseases. This review will explore non-thrombotic platelet activation in the context of allergy and the association of platelets with eosinophils, to reveal how these phenomena may lead to the discovery of novel therapeutic targets.

## Introduction

Platelets are small, anuclear cell fragments that are essential for hemostasis. Activation of platelets during hemostasis leads to shape change, α-granule, dense δ-granule, and lysosomal λ-granule release, along with surface expression of adhesion molecules and receptors, leading to platelet aggregation and clot formation (1–3). In addition to these hemostatic responses, it is now understood that platelets contribute distinct functions to non-thrombotic processes such as innate immunity to pathogens, and inflammatory disorders where there is often no associated changes in hemostatic functions of platelets. The difference in platelet function in hemostasis compared with inflammation has led to the hypothesis that there is a dichotomy in platelet activation (4), which has recently been confirmed experimentally (5, 6). It is necessary to comprehend such a distinction when analyzing the relationship platelets have with eosinophils in the context of allergic inflammation and host defense.

Platelets express an array of physiologically relevant and functional receptors that might be considered relevant to the inflammatory response in asthma and allergic inflammation, including chemokine receptors (CCR1, CCR3, CCR4, and CXCR4 receptors) (7), immunoglobulin receptors (FcγRI, FcγRII, FcγRIII; FcεRI, FcεRII, FcαRI/CD89) (8–11), toll-like receptors (TLR2, TLR4, and TLR9) (12), and certain adhesion molecules (PSGL-1, P-Selectin, and ICAM-2) (10, 13). Platelets also store inflammatory mediators in granules that can be released on activation, such as platelet factor-4 (PF4, CXCL4) (14), interleukin-1β (IL-1β) (15, 16), regulated upon activation normally T-cell expressed and secreted (RANTES, CCL5), and thymus activation regulated chemokine (CCL17) (3, 17). Therefore, platelets indeed possess the requisite components to behave as inflammatory cells, as has been demonstrated in allergic diseases including asthma, allergic rhinitis, and eczema. This short review will examine non-thrombotic platelet activation in asthma and allergic inflammation, and the association of platelets with eosinophils in these disease states, which are perhaps an inappropriate manifestation of interactions between platelets and eosinophils that occur as part of host defense against parasitic infections.

## Platelets in Asthma and Allergic Inflammation

Evidence has suggested platelet activation occurs in allergic diseases since the 1970s (18). We refer the reader to recent extensive reviews on the implications of platelet activation in asthma and give a summary below (19, 20). Following bronchial provocation of patients with asthma, there is an increased release of platelet-specific chemokines, for example, PF4 and beta-thromboglobulin (β-TG, CXCL7) (21), and mediators derived from platelets, for example, 5-hydroxytriptamine (5-HT) (22), free radical species (23), and RANTES (9). Thus, the activation of platelets *in vivo* from patients with asthma is demonstrated by the fact that *ex vivo* analysis of platelets from subjects with asthma have a diminished store of mediators, which has been linked to an apparent lack of *in vitro* responsiveness due to prior activation *in vivo*: the so-called “platelet exhaustion” (24, 25). Patients with asthma have also been reported to exhibit mild thrombocytopenia after allergen provocation (26–28) and to have shortened platelet lifespans in the circulation compared with healthy individuals (29), demonstrating that continuous platelet activation may occur as part of this disease. The mild thrombocytopenic effects observed within minutes after allergen exposure in patients with asthma suggests that platelets are recruited to the lungs (26, 28). Indeed, platelets are present in bronchoalveolar lavage fluid and in bronchial biopsies of patients with asthma. In particular, platelets are found in extravascular compartments and also in fibrous material within the airway luminal edge, indicating platelets have the ability to migrate into inflamed tissue (30, 31). Platelet chemotaxis toward inflammatory chemokines, formyl-methionyl-leucyl phenylalanine (fMLP), macrophage-derived chemokine (CCL22), and stromal cell-derived factor 1α (SDF-1_α_, CXCL12) has been demonstrated *in vitro*, through *N*-formyl-peptide (32), CCR4 and CXCR4 receptor activation, respectively (33, 34). These migratory effects may well be attributed to the fact that platelets can release enzymes contributing to movement such as cathepsin D, cathepsin E, heparinase, and β-N-acetylhexosaminidase and possess the necessary machinery to extend pseudopod-like processes and undergo actin cytoskeleton rearrangements, indicating that platelets have the mechanical capability to migrate in response to situations caused by allergic inflammation (34, 35).

### Platelets and Cellular Motility

Indeed, platelets have been shown to undergo chemotaxis directly toward allergen *in vitro* and migration through lung tissue *in vivo via* an IgE-/FcεRI-dependent mechanism (11). The induction of cellular chemotaxis by allergen appears to be atypical of how other molecules induce chemotaxis *via* GPCRs, although chemotaxis can be induced by structurally very varied molecules. It has been reported that basophils (36), mast cells (37, 38), and eosinophils (39) can undergo chemotaxis to specific allergens. These studies investigated a range of allergens and laboratory antigens and reported that cellular chemotaxis toward allergen was also IgE and FcεRI dependent (11, 37, 38), while others reported an affect *via* formyl peptide receptors (40). Clearly, the molecular mechanisms are not as well understood as chemotaxis induced by *via* GPCR activation. It is also interesting that FcεRI signaling can modify, or be modified by GPCRs, including certain chemokine receptors (41, 42). The significance of this is unknown with respect to cell motility. While it is speculative to consider whether platelet motility toward allergen might therefore modify eosinophil chemotaxis, platelets have recently been reported to be important for the intravascular crawling of neutrophils (43, 44), and platelet-dependent neutrophil chemotaxis to a range of chemokines *in vitro* (5, 45–48), and platelets might have a similar relationship with eosinophils.

### Platelets in Bronchoconstriction

It has been postulated that the platelets found within the airways of patients with asthma could propagate disease progression *via* various mechanisms. In patients with asthma, platelets can become activated following exposure to allergens, endotoxins, pollutants, and inflammatory mediators (19). The consequence of this is unknown. However, in models of allergic airway inflammation in rabbits and guinea pigs, platelet depletion prevented bronchoconstriction induced by certain substances such as bradykinin and capsaicin (49, 50), but had no effect on direct acting spasmogens such as histamine, substance P, and methacholine. Human platelets are able to produce a number of bronchoactive mediators within their granules such as histamine, serotonin, thromboxane A_2_ (TXA_2_), adenosine, 12-HETE, and cytotoxic compounds (19).

Thus, platelets that have accumulated within the lung may release stored spasmogens and affect the action of other spasmogens leading to bronchoconstriction. Furthermore, agents released by platelets might stimulate eosinophils *in situ* to cause eosinophil-dependent bronchoconstriction (19, 51, 52).

### Airway Remodeling

In bronchial asthma, chronic inflammation can alter the airway architecture that contributes to adverse effects on respiratory function. Platelet activation has been shown to persist long after the initial allergen challenge and outlasts the presence of platelet–leukocyte conjugates in the blood, displaying a potential role for platelets in chronic airway remodeling (28). Platelets produce mitogens such as TXA_2_, transforming growth factor-β, platelet-derived growth factor, epidermal growth factor, and vascular endothelial growth factor, which can have proliferative actions on cells located in the airways (19). Platelet depletion in a mouse model of allergic airways inflammation decreased epithelial thickening, smooth muscle thickening, and sub-epithelial reticular fiber deposition (53). Thus, platelet activation appears to play an important role in airway remodeling by release of extracellular matrix modifying enzymes and hypertrophic factors, causing smooth muscle hyperplasia and collagen deposition. While some of these events appeared to be independent of leukocyte activity (53), the association of platelets with eosinophil activation discussed in the following sections implies that eosinophil-associated remodeling events might be dependent on platelet activation.

## Platelet–Eosinophil Interactions Form a Part of Host Defense Against Parasite Infections

**Figure 1:**
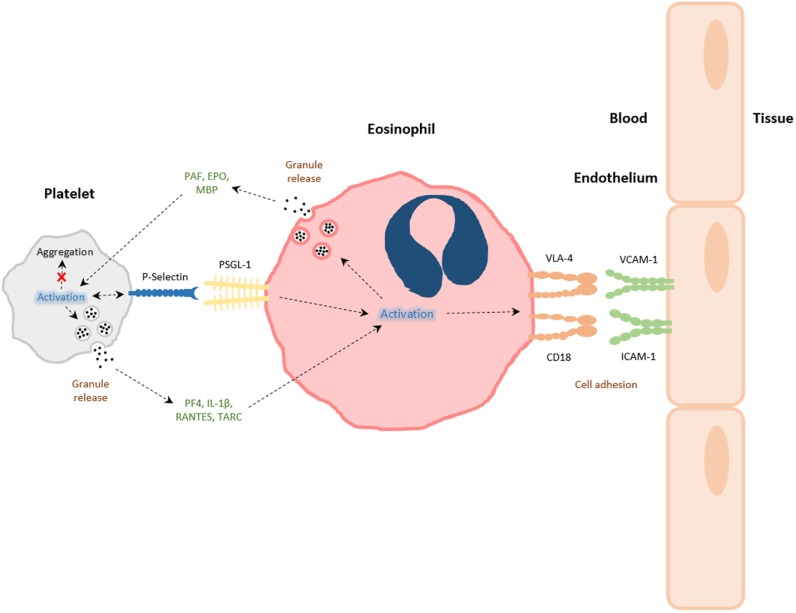
The interactions between platelets and eosinophils. Platelets and eosinophils can directly interact with one another *via* both contact-dependent (e.g., P-selectin/PSGL-1) and soluble mediator-dependent [eosinophil: platelet-activating factor (PAF), eosinophil peroxidase (EPO), major basic protein (MBP); platelet: PF-4, RANTES, IL-1β, thymus activation regulated chemokine (TARC)] mechanisms. The activation of platelets during allergic airways inflammation leads to platelet P-selectin-dependent eosinophil tissue recruitment, and activation eosinophils to express integrins very late antigen-4 (VLA-4) and CD18, or to release granule products. Granule constituents of eosinophils can likewise induce platelet activation and functions pertinent to inflammation and host defense, rather than aggregation, associated with intravascular thrombi formation.

Platelet participation is necessary during our immune defense against pathogens of bacterial, viral, and fungal origin (54), and platelet activation also occurs in immunity against parasites, for example, helminths (Schistosomiasis), protozoa (toxoplasmosis), and malaria (54–57). In particular, a role of eosinophils in helminth infections remains an important area of study (58, 59). There are therefore correlations in platelet and eosinophil activities, and that of their activation by IgE in IgE-dependent killing of schistosomes that are applicable to our understanding of the association between these two cell types in allergy (23, 58). While platelets express other immunoglobulin receptors (FcγRI, FcγRII, FcγRIII; FcαRI/CD89), the roles of these receptors in platelet responses to infection, and possible platelet effects on eosinophils have not been as extensively reported. Furthermore, it is possible that platelet–eosinophil interactions during host defense occur *via* non-immunoglobulin-associated activation pathways. We therefore discuss below a data set that is restricted to IgE/IgE receptor interactions as one example by which platelets and eosinophils can interact with each other, and which may have implications in allergic diseases. Human platelets express both high-affinity (8) and low-affinity (60) IgE receptors (FcεR1 and FcεRII or CD23, respectively). The level of expression of FcεR1 and CD23 are variable, and they are not found on all platelets from a given donor, platelet stimulation by IgE or allergen interactions can cause non-thrombotic platelet activation leading to release of cytotoxic mediators, such as reactive oxygen metabolites, free radicals, and catonic proteins (8, 61). While the physiological consequence of platelet-derived cytotoxic substances, compared to other cellular sources of the same or similar material is not known, the expression of IgE receptors on platelet surfaces was demonstrated to be important for host defense against parasitic infections as originally demonstrated in the seminal work of Capron and Joseph in the 1980s, who showed that IgE receptor stimulation of platelets was essential in killing certain types of parasites, by stimulating this generation and release of cytotoxic free radicals (8, 61). Various lines of enquiry have revealed that platelets can release chemokines in response to IgE stimulation (for example, RANTES and PF-4) that are potent toward eosinophils (62–65); and these also stimulate eosinophil free radical oxygen products (66). Platelets have been reported to release GM-CSF and thus inhibit eosinophil apoptosis, prolonging survival (67). Early studies related to the mechanism by which eosinophils engaged with *Schistosoma mansoni* revealed that selectin and Lewis X-related structures might act as coreceptors for eosinophil-mediated killing of worms (68). P-selectin mediates adhesion of platelets to eosinophils (69), and PSGL-1–P-selectin interactions between platelets and eosinophils can lead to CD18-dependent eosinophil stable adhesion (70). It is therefore of interest that there are perhaps parallels with how platelets enhance neutrophil responses to pathogens and NET formation *via* P-selectin interactions (19). Thus, it would appear that platelets have the capacity to enhance eosinophil functions against parasitic infections (see Figure 1).

The relationship between platelets and eosinophils is symbiotic. Eosinophils also release products that can potentially stimulate platelets, for example, platelet-activating factor (PAF) (71). Although the physiological consequences are unclear, an early *in vitro* investigation by the group of Gerald Gleich reported that eosinophil granule proteins, such as major basic protein (MBP) and eosinophil peroxidase (EPO) are very potent at inducing platelet α granule (β-TG), dense granule (5-HT), and lysosomal granule (β-*N*-acetylglucosaminidase) release, whereas eosinophil-derived neurotoxin and eosinophil cationic protein (ECP) had no effect (72). It was also noted that MBP and EPO stimulation of platelets was different in nature to thrombin stimulation (72). It is therefore of interest that a recent study reported that platelet aggregation was inhibited by eosinophil supernatant, and ECP in particular (73). The implications of platelet activation being induced by eosinophil-derived mediators on the one hand and inhibition of platelet aggregation to agonists by eosinophil-derived mediators on the other is difficult to interpret. It is possible that eosinophils activate or prime platelets for functions unrelated to aggregation, thereby revealing evidence for the “dichotomy in platelet function” (4). Alternatively, it is possible that different mediators derived from eosinophils promote selective functions of platelets, since there is an association of eosinophils with thrombi in patients with acute myocardial infarction (74, 75). However, the role of eosinophils in thrombosis, and a causal link to platelet activation (aggregation) has not been made, although patients with allergies have been reported to have less calcification of the major arteries, suggesting allergy might be protective (76).

## Platelet–Eosinophil Interactions in Asthma and Allergy

Eosinophilic inflammation is associated with atopic asthma, rhinitis, and aspirin-induced exacerbated respiratory disease (AERD) (77–79), therefore the interactions between eosinophils and platelets that can occur during host defense may be important for the pathogenesis of these respiratory conditions. Lellouch-Tubiana and colleagues first demonstrated in an allergic guinea pig model of asthma that eosinophil infiltration into the lung is reduced when platelet numbers are depleted in the circulation using an anti-platelet antiserum (APAS) (80). These findings were later supported by data which showed that platelet depletion *via* APAS, caused a reduction in eosinophil infiltration, and decreased hyperresponsiveness into the lungs of allergic rabbits and mice (49, 81). Subsequently, correlations between eosinophil and platelet activity have been reported in patients with asthma. Nasal wash levels of ECP and P-selectin as measures of activation of eosinophils and platelets, respectively, revealed a positive association between eosinophils and platelets, which was negatively associated with asthma-related quality of life measurements (51). Furthermore, an association of platelets with eosinophils was reported in 1992, and more recently, staining of mixed leukocyte cytospins from whole blood revealed 5–25% eosinophils attached to platelets from patients with mild or moderate asthma (82, 83), and in AERD (84) suggesting a possible role of platelets in human lung eosinophilia. While *ex vivo* measurements of circulating platelet–leukocyte (eosinophil) complexes cannot on their own be suggestive of a mechanism by which platelets influence eosinophil tissue recruitment, due to complexities of blood processing, the association between eosinophils and platelets does have functional consequences for recruitment, because eosinophils isolated from patients with asthma adhere to endothelial cells under flow conditions to a greater degree compared to eosinophils from healthy subject, and platelets promote this adhesion (85), and this important phenomenon is discussed below.

A role of platelet P-selectin-mediated events has been widely investigated in the evaluation of pulmonary eosinophil recruitment and activation, since platelets adhere to eosinophils *via* P-selectin/PSGL-1-dependent interactions, and P-selectin is important for pulmonary leukocyte recruitment (13, 69, 70, 86–90). Specifically, the adhesion of platelets and eosinophils has been investigated *in vitro*, by comparing different stimuli that activate either eosinophils, platelets or both: fMLP, thrombin, and LPS (91, 92). A blocking antibody to P-selectin and fucoidan (a non-selective selectin antagonist) was reported to suppress the rossetting of platelets around eosinophils, while abciximab (integrin αIIbβ3 antagonist) and blocking anti-L-selectin antibody had no effect (92). Furthermore, the addition of aspirin had a rather minor effect on platelet–eosinophil rosettes, while WEB2170 (PAF receptor antagonist) and MK886 (an inhibitor of FLAP) actually increased the phenomenon (92).

The specific mechanisms behind platelet and eosinophil interactions are therefore due to surface expression of adhesion molecules on activated cells (see Figure 1). In a mouse model of allergic airways inflammation, P-selectin expression on platelets was critical in eosinophil recruitment to the lung, following allergen challenge. Platelet-depleted mice that had been sensitized and exposed to experimental allergen had reduced pulmonary eosinophil recruitment after transfusion of unstimulated platelets, when compared with transfusion with stimulated platelets expressing P-selectin (13). Johansson and colleagues have reported that eosinophils taken from the blood of patients with non-severe asthma have increased levels of surface associated platelets expressing P-selectin after whole-lung antigen challenge, and these were associated with increased α4β1-integrin very late antigen-4, but not αMβ2 integrin MAC-1 expression on a proportion of eosinophils (52). β1-integrin and P-selectin appeared to colocalize on activated eosinophils, when investigated by immunofluorescence microscopy (83). The addition of soluble P-selectin to whole blood caused enhanced activation of α4β1-integrin on eosinophils and also enhanced eosinophil adhesion to vascular cell adhesion molecule-1 *in vitro* (83). Further investigations found that after whole-lung antigen challenge of patients with asthma, circulating eosinophils associated with P-selectin disappeared from the circulation, suggesting a migration of platelet–eosinophil complexes into the lungs (52). This hypothesis is supported by findings that demonstrate under flow conditions in the blood of patients with asthma, blocking antibodies directed against P-selectin, causes a decrease in eosinophil binding and clustering to activated endothelium (85). Indeed, *in situ* staining reveals platelets attached to intravascular eosinophils after allergen challenge in a murine model of allergic airways inflammation (13). Therefore, early contact-dependent interactions between platelets and eosinophils are likely to be important in subsequent eosinophil recruitment, since it is now recognized that platelet contact *via* platelet P-selectin with neutrophils is necessary for efficient neutrophil adhesion, and this has been visualized *via* intra-vital and multiphoton microscopy (6, 43, 44, 93). Such events could be initiated through the increased preponderance of platelet–eosinophil complexes. Nevertheless, along with the direct physical interactions between platelets and eosinophils, platelets can also influence eosinophil function *via* inflammatory mediator release. The platelet-specific chemokine, PF-4, is capable of accelerated eosinophil–endothelial adhesion due to upregulation of adhesion molecules (14, 94).

## Pharmacological Strategies for Modulating Platelet–Eosinophil Interactions

The development of therapeutic strategies to inhibit platelet–eosinophil interactions would be considered part of a larger research effort to modulate platelet function during inflammation (rather than solely platelets and eosinophils). Such a strategy is clearly nascent. Anti-platelet drugs (e.g., aspirin, P2Y_12_ antagonists clopidogrel, and prasugrel) used in the prevention of thrombi in patients with cardiovascular disease have not been extensively tested in non-thrombotic diseases, and especially asthma, although prasugrel was reported to have a limited (if any) beneficial effect in patients with asthma (95). Other P2Y_12_ antagonists had no effect of pulmonary leukocyte (including eosinophil) recruitment in a murine model of allergic airways inflammation (5). This would suggest that a dichotomy in platelet function exists between inflammatory responses pertinent to host defense, and platelet aggregation in response to vascular injury (4). Therefore, platelet activation and signaling pathways are likely to be separated and require a different anti-platelet pharmacy to be effective compared with established anti-platelet drugs used to inhibit the formation of thrombi. In this regard, the mechanisms of platelet activation that are necessary for pulmonary leukocyte (and eosinophil) recruitment in models of allergic and non-allergic inflammation have been shown to be platelet P2Y_1_ (5), and P2Y_14_ (96) dependent *via* signaling pathways involving Rho GTPases (RhoA, Rac-1) that are largely redundant during platelet activation in the context of hemostasis (5, 6).

Other strategies that inhibit processes associated with platelets (e.g., platelet P-selectin-dependent leukocyte recruitment) have gained traction. Bimosiamose, a small molecule antagonist to P-selectin, attenuated late asthmatic reactions following allergen challenge in mild asthmatics in a randomized, double-blind, placebo-controlled clinical cross over trial (97). Selectins are difficult structures to create effective small molecule antagonists against. Consequently, several drugs that inhibit the synthesis of PSGL-1 and therefore have the potential to suppress P-selectin-dependent eosinophil recruitment are being examined (98). These compounds have been reported to inhibit the synthesis of PSGL-1 under inflammatory conditions, rather than affecting expression at resting state, and might therefore provide an important safety benefit of not affecting the necessary immunosurveillance of the host (98). Heparin is known to inhibit P-selectin-dependent events, and a non-anticoagulant form of heparin (*N*-acetyl-de-*O*-sulfated-heparin), has recently been reported to disrupt platelet-dependent eosinophil recruitment in animal models (93).

Thus, there is a therapeutic potential in disrupting platelet–eosinophil interactions, or steps of platelet activation that have consequence on eosinophil functions, to alter the pathology of diseases associated with eosinophilia. Furthermore, an expanding volume of research is uncovering molecules whose biological pathways might in the future lead to drug development, for example, platelet-derived: 5-HT (22), IL-33 (99), Dickkopf-1 (100), and CD154 (101).

## Conclusion and Implications

Evidence now demonstrates the importance of platelets and their interactions with eosinophils in allergic disease states, with a dichotomy in their activation that is distinct to the function of platelets during hemostasis. Further research into the relationship between platelets and eosinophils may yield novel targets for drug intervention in respiratory diseases characterized by eosinophilia, for example, atopic asthma, rhinitis, and AERD, by controlling the relationship between these essential blood components.

## Author Contributions

All authors contributed to article research, writing, and editing.

## Conflict of Interest Statement

The authors declare that the research was conducted in the absence of any commercial or financial relationships that could be construed as a potential conflict of interest.
